# Influence of Athletic Sports

**Published:** 1871-05

**Authors:** 


### HYGENIC.

INFLUENCE OF ATHLETIC SPORTS ON HEALTH.—Dr. Farquharson read a paper on this subject before the Medical Society of London. Exercise, he continued, was necessary, not only to preserve the balance between body and mind, but to promote the functions. It might, however, be potent either for good or evil; and damage was often done by persons of sedentary habits indulging, without due preparation, in such exertions. Dr. Richardson had, in the "Social Science Review," drawn attention to the dangers of volunteering in this relation. The nervous system had, even in repose, a heavy strain to bear; and if to this any sudden addition were made, the destructive processes were apt to exceed those of repair. The influence of the mind was, however, necessary for beneficial exercise; and athletics seemed best to supply this combination. The Germans, French and Americans were behind us in this respect. Dr. Farquharson then showed that muscular degeneration was the result of excessive, as well as of deficient work. It was not likely that such results would follow our present system of sports; but there was reason to fear the danger of their being carried too far in our public schools, and thus checking mental progress, and dulling the clearness and sharpness of the brain. He next referred to the sports in detail. Rowing had been condemned by Mr. Skey and Dr. Richardson; but D. Farquharson endeavored to show that, from the great care exercised in picking and training crews, boating was less dangerous than these eminent authorities supposed. Gymnastics must be cautiously used when the frame was consolidated. Our public school boys or university men required valuable moral as well as physical training. It was argued that if the education of girls was eventually to be assimilated to that of men, they must also graduate in manly sports. In the treatment of the insane, active employment was most beneficial. An interesting letter from Dr. Langdon Down was read, showing the remarkable results which had obtained with idiots at Earlswood Asylum. As regarded the proper dose of exercise every one must be his own physician under ordinary circumstances; but Dr. Farquharson believed that he had seen cases of irritable heart improved by a moderate amount, aud quoted a case by Dr. Stokes in which relief from violent cardiac dyspnœa was only obtained by running. While serving in the Coldstream Guards, he had met with several cases of dilated heart in recruits from over-exertion. He condemned running; cricket he considered harmless. As to football, Dr. Farqharson's experience had been derived from Rugby; and although the game was apparently played there with great violence, an accurate list of the casualties during the past two years showed that it was comparatively harmless. One case of serious injury to the spine had occurred, and steps were taken to remove the apparently objectionable features of the game. It was not unreasonable to suppose that, although permanently serious results did not often follow that game of football in which the ball was carried by the player, necessitating falls on the head and injury to the spine, the sharpness and clearness of the mind must be blunted; and he quoted the opinion of an eminent educational authority to this effect.—*British Med. Jour.*

